# Poikilosis – pervasive biological variation

**DOI:** 10.12688/f1000research.24173.2

**Published:** 2020-09-18

**Authors:** Mauno Vihinen

**Affiliations:** 1 Department of Experimental Medical Science, Lund University, Lund, 22184, Sweden

**Keywords:** biological heterogeneity, poikilosis, noise, unifying theory, lagom, effective variation

## Abstract

Biological systems are dynamic and display heterogeneity at all levels. Ubiquitous heterogeneity, here called for poikilosis, is an integral and important property of organisms and in molecules, systems and processes within them. Traditionally, heterogeneity in biology and experiments has been considered as unwanted noise, here poikilosis is shown to be the normal state. Acceptable variation ranges are called as lagom. Non-lagom, variations that are too extensive, have negative effects, which influence interconnected levels and once the variation is large enough cause a disease and can lead even to death. Poikilosis has numerous applications and consequences e.g. for how to design, analyze and report experiments, how to develop and apply prediction and modelling methods, and in diagnosis and treatment of diseases. Poikilosis-aware new and practical definitions are provided for life, death, senescence, disease, and lagom. Poikilosis is the first new unifying theory in biology since evolution and should be considered in every scientific study.

## Poikilosis

Biological systems are dynamic and display ubiquitous heterogeneity and variation at all levels and processes. To investigate, describe and understand the entirety of variation and its significance, a new concept – poikilosis ( *poikilos, ποικιλός* in Ancient Greek for “variable” or “variegated”, and - *osis*, *-ωσις* for a suffix of “state, condition or action” – is defined. Variation is considered as an integral and important property that has numerous consequences.

*Poikilosis is inherent pervasive variation, heterogeneity and fluctuation in living organisms, populations, ecosystems, biosphere and in their components and in processes within them.*

Although PubMed lists 366,506 articles about heterogeneity (September 2020), until now there has been no general theory or framework to combine and explain the effects and properties of heterogeneity and variation. Poikilosis is discussed here in life sciences, however, it appears everywhere in nature and is relevant for chemistry and physics, as well for social sciences, humanities, economics and other disciplines. Poikilosis is an intrinsic property of all living organisms and a driver and cause of several phenomena.

Heterogeneity, and more generally poikilosis, has been largely regarded in science as noise and negative nuisance to be get rid of and to be avoided. Noise and poikilosis together affect what can be measured and perceived. Noise relates to measurements, according to the Wikipedia article for signal processing it is “unwanted (and in general, unknown) modifications that a signal may suffer during capture, storage, transmission, processing or conversion”. Poikilosis is inherent variation within biological systems, not in the measurements.

Some examples of biological heterogeneity include stochastic gene expression ^1^, DNA sequence differences that lead to >10,000 amino acid substitutions in each individual in comparison to human reference sequence ^2^, variants in one gene may be related to several diseases ^3^ and one variant can lead to different phenotypes ^4^, differences between individual genomes and in comparison to pangenome ^5^, heterogeneity of isogenic bacteria ^6^ and human cells ^7^, protein structural flexibility ^8^ and dynamics ^9^, fluctuating enzyme catalytic rates ^10^, heterogeneity in cellular machineries like ribosomes ^11^, differences in protein post translational modifications ^12^, asymmetric inheritance of degradative machineries and cell fates ^13^, protein abundance differences between individuals including twins ^14^, phenotypic plasticity ^15^, continuum of sex ^16^, incomplete penetrance of diseases ^17^, differential cellular ^18^ and individual ^19^ drug responses, diversity of gut microbiota ^20^, and predator-prey dynamics ^21^. Phenotypic and genetic variation ^22^ and ecological heterogeneity ^23, 24^ have been extensively reviewed.

**Figure 1.**
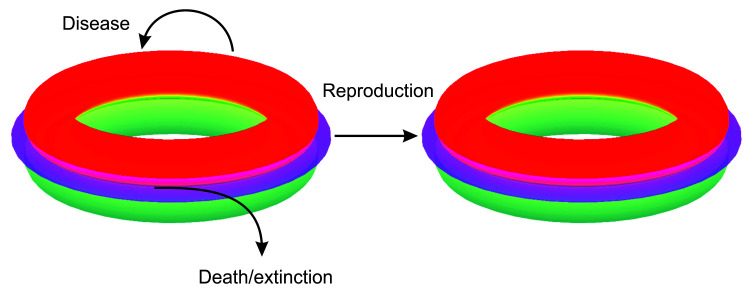
Visualisation of life, disease and death based on the principle of poikilosis. Interconnected tori in red, magenta and green indicate three of the multiple levels that interact and overlap and thereby can affect each other. Processes in living organisms are cyclic, therefore the torus shapes. Matter, energy and information flow in cyclic processes. In disease there is a deviation and by curative treatment it is still possible to return back to normal, lagom level. A large and severe deviation, which is not treated with curative care, can permanently reduce the function and adaptation capacity of the organism. Death in individual level and extinction in population level are irreversible escapes from the system. Reproduction generates new individuals that have their own interconnected levels.

Every system and process can be thought to represent its own level. Levels in here mean e.g. chemical, physical or biological entities, molecules, factors, components and their interactions in a system, but not their positions or ranks in relation to each other. In cells there are levels e.g. for genetic information, DNA, RNA and protein activity and expression, metabolic and signalling pathways. All biological processes, molecules and systems display heterogeneity and many levels are interconnected and affect each other ( Figure 1). Poikilosis has a huge number of origins of intrinsic and extrinsic type. These include stochastic processes and reactions, promiscuity and non-specificity of reactions and interactions, germline and somatic genetic variations, epigenetic alterations, erroneous repair mechanism, unspecific post translational modifications and other regulatory mechanisms, environmental effects etc. (for a review of variation generating cellular mechanisms see 25 and for protein variations ^26^). A factor can be both intrinsic and extrinsic depending on the level, e.g. what is extrinsic at the cellular level may be intrinsic for a tissue and organism.

Poikilosis emerges both actively and passively and due to intrinsic and extrinsic factors and effects. It penetrates all levels in biological systems and time wise ranges from less than a femtosecond for atom bond length and angle vibrations, to hundreds or thousands of years for individual organisms and millions of years for evolution. Poikilosis facilitates biodiversity of species, populations and ecosystems within biosphere, differences between cells, individuals and in populations, differences at genetic, molecular, structural, physiological, interindividual and other levels, and thereby a large pool of possible responses to changes in conditions.

Although biological variations have had largely negative connotations, there are some accounts of positive effects e.g. in increased cell-cell variability to cope with acute environmental stress ^27^, in mutation rate heterogeneity to increase odds of survival ^28^, in gene expression and signal transduction ^29^, in robustness of populations ^30^, and in ecological resilience ^31^. Large body of literature deals with biological noise and how to avoid and treat it, reviewed in 32. It is more fruitful to consider variation as a neutral or positive property, which is intrinsic to every system.

Poikilosis provides a new unifying theory for biology. It is compatible with many current concepts such as evolution, inheritance and selection, process regulation, continuum of pathogenicity, as wells as modern and post-modern synthesis, but it has much wider application area ranging from subatomic level to populations and ecosystems. On the other hand, poikilosis replaces some established concepts such as homeostasis and other fixed standard state conceptions.

## New definition for life

To further discuss the properties, characteristics and consequences of poikilosis we have to start by defining life. Although there is no lack of definitions for life, see e.g. 33, 34, none of them takes poikilosis fully into account. The closest has come Rollin D. Hotchkiss, who defined “Life is repetitive reproduction of ordered heterogeneity” ^35^. However, this definition is too general for our purpose and for the treatise in here it is sufficient to make the following definition:

*Life is cyclic flow of compartmentalized information, matter and energy in processes that form a self-reproductive system. Poikilosis emerges in organisms at all levels and can be selected at population level.*

Compartmentalization is essential to prevent molecules, energy and matter from being diluted to the environment. Living organisms contain highly increased or decreased concentrations of many molecules and atoms. The known life forms are compartmentalized to cells, which act as the basic units of life both for single- and multi-cell organisms.

Life forms produce, degrade and convert matter and consume and convert energy based on information that guides processes such as metabolism, catabolism, and signalling networks and development of new individuals. One type of poikilosis, genetic variation, can alter the inherited information and forms the basis for evolution. Variations have to be fixed to have a wider impact in a population. This happens via natural selection. The definition of life contains thus individual and population level components and includes enrichment of variations from individuals to population.

Information, energy and matter flow in a cyclic manner. For example, biomolecules are synthetized and degraded in cycles, and similarly genetic information in the form of polynucleotides is replicated and expressed in cycles. Information types include genetic information coded into DNA or RNA, epigenetic information, and information in signalling pathways, regulatory networks, immune system and others.

Reproduction is essential for the continuation and renewal of life forms. Life is self-reproducing and does not require outside forces for continuation. Life is penetrated by poikilosis, every living thing is unique and somewhat different from others, in its constitution, function and responses and even in its dysfunction.

Life can be visualized as concentric overlapping tori that indicate the cyclic renewing nature at different levels and the interactions of these levels ( Figure 1). Each toroid represents one level. Living organisms are in constant interaction with external and internal factors and conditions. When variations exceed acceptable levels, disease appears as a consequence. Escape from the system leads to death at individual level and to extinction at population level.

Life appears in numerous forms all of which follow the same general principles as exemplified by shared metabolic pathways and almost universal genetic code. The purpose of life is survival and continuation by adjusting to the prevailing conditions and by reproduction. An organism can adapt by adjusting itself and its responses to internal and external challenges. In populations, selection and survival facilitate evolution and adaptation.

In death, an organism loses control of variation effects, which leads to irreversible collapse of vital processes. In a multicellular organism, systems and cells die at different pace depending on their vulnerability ref. 36 and references therein.

## Effective variation

Variations and heterogeneity have a spectrum of effects. The total magnitude of a variation *V* can be presented as

E=V−R,

where *E* means effective variation and *R* the sum of reversing, attenuating, buffering and correcting factors and processes. *E* is thus smaller than *V* and can be even without any effect.

Recently functional effects of protein variants were reviewed and TARAR countermeasures were defined as biological processes that reduce variation effects (Vihinen, submitted ^37^). The model was introduced in relation to protein functional effects, but it is generic and applies to all types of effects. The T stands for tolerance, A for avoidance, R for repair, the second A for attenuation, and the last R for resistance. Similar processes apparently reduce and limit effects at all levels. Behind these five features there are a plethora of mechanisms, different at different levels.

Avoidance relates to threat management ^38^ and tolerance to ability to survive and thrive with a perturbation such as an infectious agent or genetic variation ^39^. Disease tolerance was introduced in relation to immunology to describe processes that reduce the negative impact of infections with no or even positive effect on the fitness of the infectious organism ^40^. Mounting immune system may cause more collateral damage than the tolerance of the agent. The concept of tolerance has subsequently been expanded to other effects and disease areas.

Numerous repair and rescue mechanisms actively reduce the consequences of variants at several levels. For example, genomic and dosage suppression ^41^ can restore or limit effects of genetic variants. Chaperones as general rescue molecules assist proteins to fold correctly ^42^, even if they contain a variant(s) causing somewhat defective structure and/or function. Certain activity effects can be overcome completely or partly also by promiscuity of related proteins ^43^.

Attenuation mechanisms are active or passive and include in-built resilience and robustness ^44^ as well as canalization ^45^ that returns the system back to the original path after perturbation. Robustness means resistance to intrinsic variation or environmental change. Redundancy is one of the simplest forms of attenuation ^46^. Rewiring of metabolic and signalling pathways crosslinks and reduces effects on pathways ^47^. Metabolic rewiring is in fact a hallmark for many cancers ^48^. Resistance reactions and processes actively and passively resist and reduce effects of variations and perturbations. In many diseases, genetic variants show variable expressivity or incomplete penetrance ^49^. The combined contribution of TARAR mechanisms reduces the extent of *E*. Consequences on a level may be limited to that level if the effect in other level(s) does not have a major contribution.

## Lagom: poikilosis under control

Although poikilosis is pervasive, all variations and their extents are not compatible with biological processes and systems and thereby are not acceptable. Acceptable variation ranges are here called as lagom.

*Lagom means suitable, sufficient, allowed and tolerated extent of variation at any level in an organism, population, biological system or process.*

Lagom is a central concept in Sweden and in Swedish, where it means sufficient, not too much not too little, in other words balanced and just right. Lagom carries the connotation of appropriateness, but not perfection.

**Figure 2.**
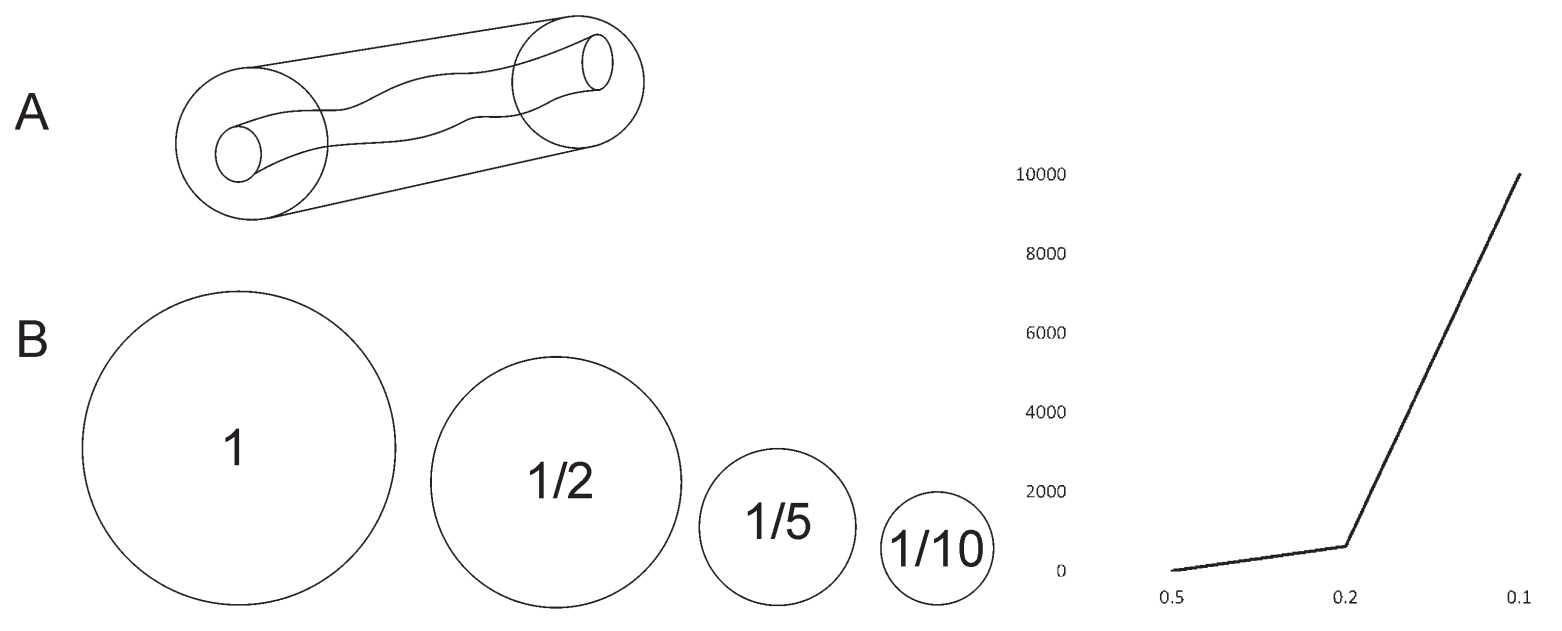
Visualisation of lagom, variation zone, and costs for regulation. **A**. Part of one torus indicating the possible range of variation (outer tube) and variation zone (inner shape) within a level. Variation zone indicates the dynamically changing lagom level of variation. **B**. Cost of feedback control the efficiency of which is in quadric power. The smaller the allowed variation, the larger the cost. Variation within lagom extent is costless, whereas set point-based homeostasis would mean extensive cost. The circles indicate the reduction of heterogeneity from original situation to half, one fifth and to one tenth. The increasing control costs are shown to the right.

Variation zone is an artificial reconstruction of the lagom extent of variation for a poikilosis component. Variation zones are dynamic, both the positioning of the zone and extent of variation within it are variable and dependent on situation, environmental condition etc. see Figure 2A. The large pipe indicates the universe of possible variation within one level, while the smaller shape indicates dynamic lagom variation. TARAR mechanisms limit and reduce consequences of variations to lagom extent in normal situations.

Biological systems and organisms contain many regulated processes. Well known examples are human blood glucose level and body temperature. Even these processes display heterogeneity. Recently, single-cell studies have revealed wide variations in many systems that previously were anticipated to be homogeneous ^50, 51^.

Homeostatis ^52, 53^, and its updated versions homeorhesis ^54^ and allostasis ^55^, is based on the concept of a static ideal state, a “set point”, to which the system is actively returned by negative feedback loops after any change or perturbation. Homeostasis and other set point-based conceptions are not compatible with poikilosis. Poikilosis restricted by lagom performs similar regulation but is conceptually exactly opposite idea of variation and its restriction.

Life does not strive towards perfection, instead at lagom i.e. sufficient and relevant reactions and responses. As an example, enzymes are essential catalysts that facilitate reactions spontaneous rates of which are far too slow for living systems. They can increase reaction rates up to 10 ^26^ fold ^56^, although most enzymes are much less efficient, since there is no need for the highest reaction rate and nature does not optimize reactions and systems beyond sufficient performance. Similarly, enzymes and reactions are not entirely specific. The rigid lock and key model ^57^ does not describe reality of biological interactions, since enzymes are promiscuous and process a range of substrates and may have several activities. The goal of life is survival, not perfection, which means that it is relevant and sufficient to have lagom activities and processes, not more efficient. Thus, there is no selection pressure to increase activity or functionality beyond relevant and sufficient extent or to regulate a system beyond what is pertinent for it.

Excessive variation is harmful and e.g. fetuses with too large variations are not viable and cause miscarriage in higher organisms ^58^. On the other hand, too restricted or limited variation has negative consequences. Endangered species degenerate because their pool of genetic variation is too restricted ^59^. Similarly, consanguinity leads to limited variation and enrichment of genetic defects and diseases in a population ^60^.

## Redefining disease and death

Non-lagom variations have negative effects which affect interconnected levels. The consequences do not remain at one level, unless the effects on the other level(s) are within lagom extent for that level. Once the effects are large enough a disease emerges. Too large a variation has multilevel effects first locally but can evolve and spread to become systemic. A new definition for disease is warranted.

*Disease is a systemic deviation, defect or failure due to non-lagom variation leading to cumulative consequences in several levels.*

This is related to but different from naturalist definitions including biostatistical theory ^61^.

Phenotypic heterogeneity within a disease can hamper accurate diagnosis as the phenotype, signs, symptoms, and laboratory values could match with several diseases. Since many levels are connected to others, similar effects and signs can originate from different primary variations in different levels. Disease consequences and symptoms vary according to non-lagom variation extent and cause, as well as due to progression, duration and severity of the condition. A pandemic is a disease at (sub)population level occurring when a large number of individuals has the same vulnerability for an infectious agent.

The extent of multilevel effects has wide individual variation range. In the case of smaller variations, the system returns back to lagom level relatively quickly and without major consequences. Larger deviations may lead to damage of some kind and possibly impair or reduce the functionality and adaptability of the system or organism. In extreme cases of most severe conditions, there is a domino-like effect spreading effects to new levels and eventually leading to death. The systemic extent varies markedly between diseases and between individuals suffering of the same disease. Low grade inflammation is an example of mainly tolerated condition, which however is a risk factor for a number of diseases.

*Death is caused by excessive multilevel variations that irreversibly collapse vital processes and functions, and spread to become systemwide.*

Curative treatments aim to reconstitute the system back to lagom extent of variation on all the affected levels. Such treatments are available just for a small fraction of known diseases, therefore, many conditions are treated with palliative care to reduce the extent of variation effects. Multimorbidities are challenging to diagnose and treat since many connected levels, systems and processes are simultaneously affected.

Organisms change gradually during time. Senescence *per se* is not a disease but can contribute to many diseases. It can be defined as follows:

*Senescence originates from lifetime accumulation of variations in an organism. Although many of these variations are corrected, attenuated, resisted or tolerated, the increasing burden of variations eventually leads to permanent non-lagom effects and costs for the individual.*

Depending on the combination of variations, senescence-related effects and their severity vary between individuals. Persistent variations cause chronic effects and become a burden.

As shown above, poikilosis is an integral component in medicine, therefore diagnosis and treatment will require a new line of thinking how to define diseases. The changes may not have to be extensive since some aspects of variation and heterogeneity are already taken into account in certain specialities. Many-valued logic with more than two or even with infinite number of truth values has been applied to diagnosis and other applications in some diseases ^62, 63^. One way to take heterogeneity in diseases into account is to apply pathogenicity model that describes the continuum of a disease as a joint outcome of three factors: extent, modulation and severity ^64^. Implementation of the pathogenicity model will facilitate the comprehension of variation as well as its consequences for making diagnosis, decision on treatment and e.g. pharmacogenetics and patient stratification ^65^.

## Cost for poikilosis is moderate

The costs of poikilosis are estimated here from three perspectives. First, effective variation restricts and reduces costs of many variants and lagom means that a range of variations is accepted without extra costs. Second, poikilosis reduces the generated Gibbs free energy in living organisms to nominal level. Third, maintenance of a system at lagom level bears only modest costs.

Cost *C* of a variation at level *i* can be formulated as

$$C_{i}=E_{i}-L_{i}$$

where *E _i_* indicates the effective variation and *L _i_* is lagom extent of variation at the specific condition. *C _i_* < 0 means that the cost is within lagom range at the level and thus variation is acceptable. When *C _i_* > 0 the variant has a net cost at a level and may affect other levels. The total cost *C _tot_* of a variation is the sum of its expenses at all affected levels. When the cost is too high, lagom level is exceeded. Lagom extent on a level varies dynamically depending on the situation in the system ( Figure 2A). Biological systems and processes, such as pathways, cells, organisms and populations, favour low cost solutions to perturbations.

Lagom variation extent reduces the net costs of biological systems as variations within the variation zone, where variation lies for most of the time in normal situations, do not bear any extra cost. Utmost performance, perfect symmetry or phenotype would require extensive costs in surveillance, repair and other expenses. Life and nature do not gain any benefit from perfection. For example, fluctuating asymmetry has often been considered as a developmental instability and deviation ^66^, however there is no benefit for perfect symmetry. Similarly, protein activity has just to be sufficient, not at highest possible speed, specificity etc.

Organisms are open systems, thus increased order caused by life is not against the second law of thermodynamics. According to this law the total entropy in a closed system remains the same or increases, but does not decrease, over time. Organisms are not closed systems, they are part of their surroundings. They input free energy and export entropy in the form of waste and heat. As life requires sufficient and not perfect i.e. lagom organization, the effect on entropy, more precisely on Gibbs free energy, is not excessive. In comparison, homeostasis would require substantial contribution to free energy to keep the system at a set point.

The magnitude of the Gibbs energy difference due to life could be analogous to stabilizing effects in globular proteins, where there is only a small 3-15 kcal/mol difference between the folded and unfolded states ^67^, an amount that equals the sum of just a few bonds and interactions.

Homeostasis means a stable constant state that has to be actively maintained with negative feedback control. To implement such a system, extensive and costly monitoring and regulation is needed. Even the most effective biological feedback circuits reduce the variation with the fourth root of the number of signalling events (number of control molecules) ^68^. To reduce the standard deviation of variation to half requires 16-fold (2 ^4^) excess of control molecules ( Figure 2B). More stringent regulation by 10-fold would demand at least 10,000 i.e. 10 ^4^ times excess of the monitoring molecules. Thus, set point-based control mechanisms (homeostasis, homeorhesis, allostasis, proteostasis etc.) are not feasible due to the excessive cost for the production and maintenance of the control machinery. Maintenance of poikilosis at lagom level within variation zone introduces only a low or modest cost ( Figure 2B) and is energetically and cost-wise feasible but it still facilitates the required control.

## Correlation to evolution and survival

Poikilosis is fully compatible with the evolutionary theory and in fact it facilitates evolution as it provides states from which to choose fitted combinations by natural selection. It provides material for selection, but not only genetic variations.

The concept of the survival of the fittest actually means the survival of the individuals which have a relevant combination of tolerance, resistance and attenuation and suitable lagom variation. It does not mean that the strongest or fastest or any other property described with a superlative would be the fittest. Large enough poikilosis in a population guarantees the survival of at least some individuals in all but the most drastic changes in environmental conditions. However, poikilosis has to be kept at lagom level since in a stable situation excessive or too limited variation would have negative effects. Variants deviating further away from neutral zone for optimal protein stability (i.e. lagom) either destabilize or stabilize the protein and reduce the fitness of the organism ^69^. Which genetic and possibly epigenetic variants are essential for adaptation depends on the situation. Founder variants may not have been the most optimal alterations for survival but were enriched due to being present in the population when needed.

Which variants are fixed in a population depends on many factors, population size being an important one. Natural selection, genetic drift and genetic variations are weak evolutionary forces at generation level, their strength comes over extended time frames ^70^. Protein structural epistasis, where a compensatory variant rescues and saves from harmful effects of a variant ^71^, has a strong impact on evolutionary trajectories.

Mathematical formulation of evolution as a differential equation of motion revealed that evolution can be described with the second law of thermodynamics as an energy transfer process. Based on this model, natural selection favours variants that lead to faster entropy increase in the system ^72^. Thus, the most probable path of evolution follows the steepest energy descent.

## Poikilosis-aware study design, experimentation and data analysis

By considering poikilosis more realistic analysis, prediction and modelling of biological systems can be achieved. Changes will be required to concepts, experiments, analyses, predictors, models and simulations to fully include poikilosis as an intrinsic feature of systems instead of trying to get rid of unwanted “noise”. Full consideration of poikilosis requires five steps: understanding the investigated phenomenon and variation and its lagom extent, knowing and testing effects of noise, detailed description and annotation of experiments, experimental design including poikilosis, and data analysis and interpretation that are aware of poikilosis.

The first step for including poikilosis is understanding the investigated phenomenon or process and variation within it. Variation Ontology (VariO) is an example of systematics for describing variation within a knowledge domain ^73^. It was designed to describe effects, consequences, mechanisms and types of variations at DNA, RNA and protein levels. Detailed explanations and examples have been published for protein. RNA and DNA variants ^74– 76^. This kind of framework lends power for describing the type, extent and context of observed poikilosis.

Measurements and experiments contain components of both poikilosis and noise. Second, it is essential to discern the effects of noise that confound the true signal from experiments. How that should be done varies for the investigated systems and used measurement instruments as the signal to noise ratio may not be linear over the investigated measurement range and therefore may command for use of advanced approaches. Numerous factors contribute to uncertainty of measurements.

To facilitate true comparisons of experiments, the third step demands very detailed description of the used methods, instruments, reagents, samples, experimental conditions and other details. These annotations have to be much more thorough than currently customary in many scientific journals. Several best and good practice guidelines and minimum information requirements have been published to describe various aspects of experiments, many of which are available at FAIRsharing ^77^. Systematics and harmonization of these annotations are of utmost importance to facilitate reproducibility and analyses and comparisons of data sets from different laboratories and consortia.

Fourth, poikilosis has to be included already into experimental design and conduct. Noise and poikilosis jointly affect studies and have to be divided into components. It is likely that in many studies the number of replicates has to be increased compared to the current approaches to chart the extent and characteristics of poikilosis and noise. To confirm the observations, it is recommended to repeat the experiments in another independent but related system, like cell line, population or habitat, whatever is relevant for the study.

In the fifth stage, data analysis has to be geared towards poikilosis. Some steps have already been taken to consider poikilosis as an intrinsic component of systems. Examples include probabilistic trait loci in genetics ^78^, cell population modelling in biology ^79^ and pathogenicity model in diseases ^64^. However, it is obvious that new physical and mathematical models are needed in many fields to fully capture the extent and significance of variation ^80^.

Traditionally, many studies have been based on metrics for the point estimates of population average for investigated items and thereby completely neglecting poikilosis. Gough *et al.* ^81^ discuss metrics of heterogeneity in regard to the shape (modality) of the distribution, extent or diversity, and the tails of the distribution. They list approaches that have been used to address these aspects including univariate, Gaussian statistics, Gaussian models and nonparametric statistics, entropy, spatial, temporary and combined metrices. Squared coefficient of variation and Fano factor have been applied in some areas, however have assumptions that do not hold with real data ^25^. Noise filtering methods, like Kalman filter, and information measures including Shannon entropy and Gini index, quantify heterogeneity and with suitable data could be used for studies of poikilosis. The shape of the distribution and its visualisation inform about the type of modality and tails (kurtosis and skewness). Current methods for the analysis of the overall distribution and tails each have their pros and cons but do not fully cover poikilosis.

The majority of available prediction methods in many fields are binary in design without consideration of the continuum of variation. This is the case also in tools for genetic variation interpretation. Most variation tolerance/pathogenicity predictors consider two states, benign and pathogenic. More realistic approaches are needed. For example, PON-P2 ^82^ predicts variants in three categories, benign, pathogenic and of unknown or variable effect. The first generic variant severity predictor PON-PS ^83^ has also three categories for benign, mild and severe phenotypes. Whether more detailed grading is required, depends on the application, however, the number of predicted classes is often limited by the amount of known experimentally validated cases.

## Connotations and implications of poikilosis

Although literature on heterogeneity is voluminous, there are not many studies that have tried to organize or provide theory for it. Heraclitus of Ephesus had as a cornerstone of his philosophy *panta rhei (*πάντα ῥεῖ), meaning everything flows and changes i.e. pervasive flux, change or becoming (quoted in Simplicius' Commentary on Aristotle's Physics). The idea of pervasive cellular variation was presented by Elsasser ^84^, and more recently variation was divided into three categories: population, spatial and temporary heterogeneity ^81^, but there are many more levels as shown above.

Evolution has been the only unifying theory in biology. It was subsequently combined with genetics to form modern synthesis. More recently, efforts have been made for post-modern or extended synthesis by including e.g. epigenetics and evo-devo aspects. Poikilosis is compatible with these central theories and goes much wider and beyond inherited traits. Poikilosis is a generic concept describing and based on variation at all possible levels in organisms and systems.

Evolution is facilitated by poikilosis. The inherent heterogeneity of all processes and levels including genetic variation means that populations contain wide spectra of variations and states from which to choose the fitted ones, if needed. The old statement of the survival of the fittest could be rephrased as survival of the variable meaning that traits that facilitate adaptation to new situations are selected. Poikilosis provides variations for organisms to adapt, for phenotypic heterogeneity, and it facilitates strategies as bet-hedging ^85^ and eventually it provides material for natural selection and evolution.

During recent years, reproducibility of scientific studies and their results have been brought up since many investigations published even in the most prominent journals could not have been repeated ^86, 87^. There are numerous reasons for the irreproducibility, the lack of consideration of inherent poikilosis being one of them. Poikilosis should be taken into account already in the design of experiments, in conduction of studies and analysis and interpretation of results. Recent suggestion to (again) retire the concept of statistical significance ^88^ has emerged due to erroneous description of differences and their meaning. Knowing the intrinsic poikilosis of a system is a prerequisite for understanding differences. As example, very large genetic, transcriptional, translational and turnover rate differences were noted in widely used cell lines, in 27 strains of the breast cancer cell line MCF7, and 14 stocks of HeLa cells from different laboratories, respectively ^89, 90^. Cell lines show marked differences also for cancer drug responses ^89^. Single cell study of human fibroblasts, which are considered as a very homogenous cell population, revealed large variations in three dimensional global genome organization ^91^. Thus, studies even on standard systems without considering poikilosis at many levels are likely to fail or at least provide somewhat misleading outcomes.

Related to reproducibility and overall reporting of poikilosis, scientific literature has to start to demand detailed descriptions of conducted studies as well as of the investigated samples and systems. Reporting guidelines are available e.g. for systematic reviews and meta analyses ^92^, prediction method description and performance assessment ^93, 94^, multiple sequence alignments ^95^, and more than 200 guidelines for health reporting have been reviewed ^96^. Existing systematics should be used, or developed if not available. However, even when guidelines and standards are available, they are often not followed or applied only selectively. By following FAIR principles ^97^ the sharing of data will facilitate confirmatory and repeated experiments. Combined with open access to data principle soon to be implemented in several countries, reproducibility will be increased and the extent of poikilosis can be revealed.

Increasing evidence in scientific literature supports individual parts and areas of the theory of poikilosis. Some of these are mentioned above, further examples will be given in here, but there are far too many to cover but a small fraction of the existing literature. However, none of these studies has taken poikilosis fully into account.

Nature takes benefit of variations in many ways and certain processes have evolved to generate huge variability. Recognition molecules of adaptive immune system, B- and T-cell receptors and antibodies, are produced by a specific variation generation machinery. The combination of V(D)J recombination, somatic hypermutation and class switch recombination generates a vast array of molecules with different binding sites to detect foreign compounds and organisms. There are in the order of 10 ^10^ different possible recognition molecules. Somewhat similar outcome, but in smaller scale, is produced by errors in transcription, translation and expression machineries, especially under stress situations. Alternative splicing, initiation and termination are among processes that can generate numerous mRNA and protein forms. *Drosophila melanogaster Dscam* gene for Down syndrome cell adhesion molecule has totally 38,016 possible splicing isoforms by different combinations of its 24 exons ^98^. The protein has two alternative transmembrane fragments, the other variants appear in three immunoglobulin domains.

Variation in cell populations can enable information coding and transfer as well as rapid responses based on crowd control, as only some cells in a population may detect a signal but they can launch a coordinated response ^99^. Diversity of cell states in a population makes it possible to adapt to environmental changes. This is called bet hedging and means that the fitness of the population is somewhat decreased in stable conditions, but significantly increased in stressful conditions ^85^. Synchronization of cell populations to act as on-off switches may not be the optimal strategy to respond, since dose-dependent responses are smoother ^99^. Still another cell level process where heterogeneity is beneficial is fate plasticity of stem cells ^100^.

Diseases are systemic deviations due to non-lagom variation affecting several levels. By considering poikilosis in medicine, the continuum of the conditions becomes apparent. As pathogenicity model ^64^ indicates, similar disease states in patients can be sums of different disease components. Curative medicine aims at returning the system back to lagom variation extent while palliative care reduces the effects of variation but does not lead to full recovery. Poikilosis-aware strategy has been presented for diagnosis, prognosis, patient stratification and drug development for COVID-19 ^65^.

Cancer is an example of a disease where variations exceed lagom at many levels. Cancers indicate also how robust and resilient organisms are even for excessive variations. Despite even more than 1 million genetic variants e. g. in lung cancer ^101^ affecting multiple levels, even the most aggressive forms of cancer require extended time to finally collapse the systems.

Poikilosis explains differences also in drug treatments. Individuals respond differently due to their heterogeneity indicating the need for personalized medicine ^18, 19^. Similarly, adverse drug reactions have a wide spectrum as individuals react differently.

Many biological and bodily functions and processes are tightly regulated, but instead of a fixed set point each system display some variation. Currently, many disease diagnoses and treatments are based on the idea of homeostasis despite of its flaws. Poikilosis is conceptually totally different although many outcomes are similar from the surface. In homeostasis the system strives to keep some ideal stable state. Homeostasis and related concepts are not feasible since they would require extensive energy and other resources for monitoring and controlling, especially since the efficiency of feedback control systems is very low ^68^. Synthesis of monitoring molecules in vast excess to controlled compounds would be extremely costly and require substantial portion of energy available for an organism. Dynamically adjusted poikilosis kept at lagom level facilitates sufficient control at reasonable and acceptable cost ( Figure 2B).

## Conclusions and prospects

Poikilosis means constant changes that in biology are controlled at lagom level. Lagom fluctuates on the variation zone, which changes dynamically depending on the situation and even the extent of lagom variation varies in time, space and due to perturbations. Lagom is relevant for biological systems and processes instead of perfection, which does not provide any benefit, but which would require extensive control machinery and costs in many ways.

Poikilosis as a concept is in certain extent analogous to junk DNA, as the non-coding part of genomes was still recently called due to ignorance of its meaning, function and purpose. To reveal the full importance of poikilosis, something similar to The Encyclopedia of DNA Elements (ENCODE) project ^102^ for the noncoding genome would be needed. The project could start by analysing large volumes of existing information and learn about poikilosis, its extent and consequences in systems of interest. Reanalysis of obtained results and observations could be the solution at some instances or could indicate how more comprehensive studies should be performed.

Poikilosis is not restricted to scientific endeavours. It penetrates also human culture. Many forms of art are based on heterogeneity and take benefit of it. For example, symphonies are based on variation and development of a theme. Many artists generate variations of the same central ideas and motifs throughout their careers, cf. self-portraits of Vincent van Gogh or female figures in Pablo Picasso’s paintings.

Awareness of poikilosis could hopefully contribute towards acceptance of differences between people. Wide adaptation of the concept of poikilosis could increase understanding and acceptance of e. g. social, sexual and ethnic variation. Evolution has in addition to its biological significance had wide effects including social and cultural aspects and poikilosis has a potential to make similar contribution.

## Data availability

### Underlying data

No data are associated with this article.
