# Supplementary material for: Poikilosis – pervasive biological variation
Source: F1000Res. 2020 Sep 18;9:602. Originally published 2020 Jun 12. [Version 2] doi: 10.12688/f1000research.24173.2 (PMC7463298; doi:10.12688/f1000research.24173.2)
Supplement: Peer review history

# Peer review: Reviewer response for version 1

**DOI:** 10.5256/f1000research.26664.r68772

**Published:** 2020-09-01

**Authors:** Xavier de la Cruz

**Affiliations:** 1 Research Units in Clinical and Translational Bioinformatics, Vall d'Hebron Research Institute (VHIR), Barcelona, Spain

This is an interesting contribution in which the author presents and develops the concept of poikilosis which unifies the different types of heterogeneity observed in the different complexity levels of biological organisms. A variety of examples of poikilosis are discussed in the text and, importantly, life, disease and death are redefined in terms of this central concept. This work, presented as an Opinion Article, is of interest to those interested in understanding how effects repeating through the biological hierarchy can be unified. I have a few comments on some specific issues that, from my point of view, require clarification or minor action.

- In p2, the author mentions that 'Some examples of biological heterogeneity include uncertainty of positions of electrons...' I believe that this example, considering the reference given, is closer to physics rather than biology. I feel that its extreme nature dilutes the value of the examples listed afterwards, more related to biological studies. 
- In p5, the author states ‘Life does not strive towards perfection, instead at lagom i.e. sufficient and relevant reactions and responses.’ Finalism slipped into the sentence, clashing with the idea of life as a physico-chemical process.
- In p6, the sentence 'Second, poikilosis reduces the generated Gibbs free energy in living organisms' expresses the existence of a relationship between poikilosis and a thermodynamics variable, the Gibbs free energy. I believe that, even if short, an explanation should be provided justifying relationship, because it is not easy to derive from the definition of poikilosis.
- In the same line, in the section 'Correlation to evolution and survival', it would be valuable to briefly mention the relationship between poikilosis and fitness, a key parameter in evolutionary studies that is related to free energy change upon mutation in proteins (DePristo et al., Nature Rev. Genet, 2005 ^1^).
- In p6, the sentence 'Perfect performance or phenotype would require...' suggests the existence of perfect phenotypes. This concept is not very clear and may have undesired implications in a work where all levels of biological complexity are considered, included the population level. I suggest that the idea of perfect phenotype is either clarified or discarded.

Is the topic of the opinion article discussed accurately in the context of the current literature?

Yes

Are arguments sufficiently supported by evidence from the published literature?

Yes

Are all factual statements correct and adequately supported by citations?

Yes

Are the conclusions drawn balanced and justified on the basis of the presented arguments?

Yes

Reviewer Expertise:

Clinical and Translational Bioinformatics

I confirm that I have read this submission and believe that I have an appropriate level of expertise to confirm that it is of an acceptable scientific standard.

## References

[1] : Missense meanderings in sequence space: a biophysical view of protein evolution. *Nat Rev Genet*.2005;6(9) : 10.1038/nrg1672 678-87 10.1038/nrg1672 16074985

# Peer review: Reviewer response for version 1

**DOI:** 10.5256/f1000research.26664.r67620

**Published:** 2020-08-11

**Authors:** Emil Alexov

**Affiliations:** 1 Computational Biophysics and Bioinformatics, Department of Physics and Astronomy, Clemson University, Clemson, SC, USA

The manuscript reports a novel approach to deal with dynamics and heterogeneity in biological systems. This new approach is called “Poikilosis” and it is defined by the author as “inherent pervasive variation, heterogeneity and fluctuation in living organisms, populations, ecosystems, biosphere and in their components and in processes within them”.

  Major comment:

 Throughout the manuscript “molecules” and “matter” are considered to be different entities. The same is about “atoms” and “matter”. However, “atoms” and “molecules” are the building block of the matter.

 Minor comments: 

1. Figure 1 is not informative. Perhaps it can be reconsidered and the effect of disease/curative treatment shown in some cartoonish presentation. Another note, the reproduction (presumably an offspring) is shown with smaller tori, which can be wrongly interpret as lower complexity than in the parent.
2. p. 2: “Every system and process can be thought to represent its own level.” What level?
3. P.3. The paragraph starting with “Information, energy and matter flow…”. Epigenetic  information, and information in signalling pathways, regulatory networks, immune system are encoded in DNA. Why they are considered to be independent source of information?

Is the topic of the opinion article discussed accurately in the context of the current literature?

Yes

Are arguments sufficiently supported by evidence from the published literature?

Yes

Are all factual statements correct and adequately supported by citations?

Yes

Are the conclusions drawn balanced and justified on the basis of the presented arguments?

Yes

Reviewer Expertise:

Personalized Medicine

I confirm that I have read this submission and believe that I have an appropriate level of expertise to confirm that it is of an acceptable scientific standard.
